# UV photodamage pathways and the evolutionary selection of thymine over uracil in early genetic systems

**DOI:** 10.1073/pnas.2615278123

**Published:** 2026-08-03

**Authors:** Keyvan Khosh Abady, Negar Karpourazar, Peter M. Rentzepis

**Affiliations:** ^a^ Department of Electrical and Computer Engineering, Texas A&M University, College Station, TX 77843

**Keywords:** UV-induced DNA damage, cyclobutane pyrimidine dimers, nucleobase photochemistry, molecular evolution, origin of life

## Abstract

The evolutionary selection of thymine over uracil in DNA presents a paradox from a photostability perspective, since thymine is found to be more susceptible to photodamage from the intense UV radiation present on the early Earth. This study addresses this paradox by providing a comparative spectroscopic analysis of thymine and uracil under controlled 265 nm UV irradiation. To that effect, we used steady-state absorption, fluorescence, and Raman spectroscopy to quantify and compare the photochemical behavior and damage kinetics of the two nucleobases. Our results show that thymine is inherently more photoreactive than uracil, exhibiting a faster overall rate of photodamage, a broader absorption band that increases spectral overlap with the primordial UVC spectrum, and a redshifted absorption maximum located in a spectral region where solar intensity was substantially higher. However, kinetic analysis of lesion pathways reveals that thymine forms irreversible (6-4) photoproducts at a significantly lower rate than uracil, instead directing UV-induced damage primarily toward the reversible cyclobutane pyrimidine dimer pathway. These findings support the hypothesis that canonical nucleobases were evolutionarily optimized not to minimize photodamage but to localize damage within the bases and direct lesion formation toward reversible pathways amenable to nonenzymatic self-repair.

## Significance

Why DNA uses thymine instead of uracil remains photochemically unresolved, since thymine is more susceptible to ultraviolet (UV) damage. Resolving this paradox is central to understanding how life emerged and persisted under intense early Earth UV radiation. Using absorption, fluorescence, and Raman spectroscopy under UVC irradiation conditions relevant to primordial Earth, we show that although thymine is more photoreactive, it preferentially channels UV damage into reversible lesions amenable to nonenzymatic self-repair. These findings support the concept that nucleobases evolved as “molecular sunscreens” that protect genetic integrity by localizing and controlling damage rather than avoiding it. Our spectroscopic measurements establish a reference framework that enables sensitive detection and quantification of UV-induced damage, with applications in pathogen diagnostics and UV disinfection monitoring.

DNA is a double-stranded helical polymer that carries the genetic instructions essential for the development, functioning, and reproduction of living organisms, preserved primarily in the nucleus of eukaryotic cells or the nucleoid region of prokaryotic cells (1–5). In contrast, RNA is typically a single-stranded polymer that plays diverse roles within cells, including protein synthesis (mRNA), gene regulation (miRNA), and enzymatic functions (ribozymes). The monomers of RNA are nucleotides similar to those of DNA, but differ in two fundamental ways: They contain uracil instead of thymine as one of the nucleobases, and their sugar component is ribose rather than deoxyribose (6–11). Given the complex molecular structures of DNA and RNA, as well as their intricate roles in essential biological processes, these molecules are susceptible to various types of functional errors and structural damage that compromise genomic integrity and cellular functions.

**Fig. 1.**
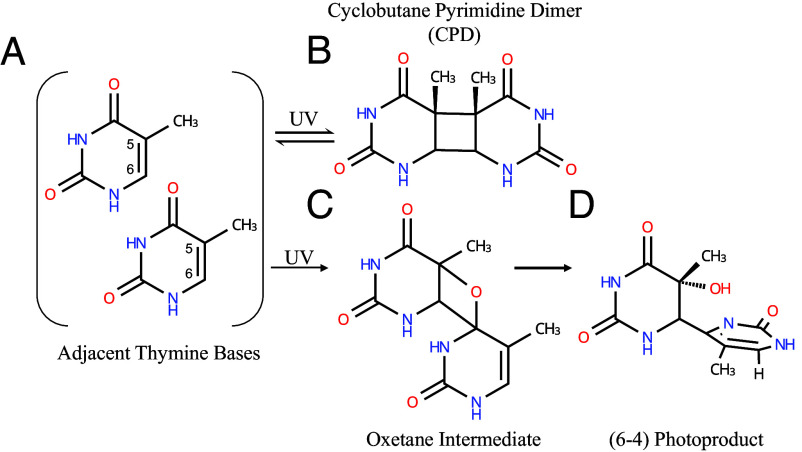
Schematic representation of the formation mechanisms of UV-induced CPD and (6-4) thymine photoproducts. (*A*) Two adjacent thymine bases on the same DNA strand. (*B*) CPD formed through [2+2] cycloaddition between adjacent C5=C6 double bonds following UV absorption. (*C*) Transient oxetane intermediate generated during the formation of a (6-4) photoproduct. (*D*) Final (6-4) photoproduct characterized by a covalent bond between C6 of one thymine and C4 of the adjacent thymine.

Among the various agents capable of inflicting such damage upon DNA, ultraviolet (UV) radiation is recognized as a potent genotoxic factor. Specifically, UVC radiation (200 to 290 nm) is most efficiently absorbed by nucleobases and is the most potent inducer of photodamage (12–15). Direct absorption of UV radiation by DNA molecules excites electrons from the π-bond of the C5=C6 double bond in the thymine base to the π*-antibonding orbital. In the excited state, the C5=C6 double bond dissociates, forming two separate reactive sites with unpaired electrons. If two adjacent thymine bases on the same DNA strand are in close proximity (Fig. 1*A*), the excited thymine can interact with the C5=C6 double bond of a neighboring ground-state thymine. This interaction leads to the formation of a four-membered cyclobutane ring, covalently linking the two bases to form a cyclobutane pyrimidine dimer (CPD; Fig. 1*B*), which is the most common type of UV-induced DNA photoproduct.

The second most frequent type of UV-induced direct DNA damage is the formation of (6-4) photoproducts. The formation of these lesions involves the absorption of UVC radiation by the C5=C6 double bond of one thymine, inducing a π→π* transition, while the carbonyl group (C4=O) of the adjacent thymine undergoes an n→π* transition. These concurrent electronic excitations render the C6 of one thymine nucleophilic and the C4 carbonyl carbon of the adjacent thymine electrophilic, driving the formation of a covalent bond between them. This is followed by a secondary linkage between the carbonyl oxygen and C5 of the neighboring thymine, producing a transient oxetane intermediate (Fig. 1*C*) that rapidly rearranges into the final (6-4) photoproduct (Fig. 1*D*), which is characterized by a covalent bond between C6 of one thymine and C4 of the adjacent thymine. The formation of CPD and (6-4) photoproducts between adjacent thymine molecules disrupts the hydrogen bonds between bases of complementary DNA strands and consequently can interfere with crucial biological processes such as DNA replication and transcription, potentially leading to mutations or cell death (16–26).

In RNA, both CPD and (6-4) photoproduct formation represent biologically significant photodamage induced by UVC irradiation, following a mechanism similar to that in DNA but with two key structural differences. First, the single-stranded nature of RNA reduces its structural rigidity and results in a more flexible backbone, which prevents adjacent pyrimidine bases from consistently aligning in the precise geometry required for photoproduct formation. Second, RNA contains uracil instead of thymine, which lacks the 5-methyl group present in thymine. This methyl group enhances base stacking via CH–π interactions and van der Waals contacts with adjacent bases, stabilizing tightly packed, face-to-face arrangements that favor overall photoproduct formation. The absence of this methyl group in uracil reduces stacking strength, which together with RNA’s inherently more flexible single-stranded backbone disfavors the precise geometries required for efficient photoproduct formation. As a result, overall UV-induced photodamage occurs less efficiently in RNA than in DNA. However, despite the biochemical and structural differences between thymine and uracil, their relative photostability has not yet been quantitatively established through direct spectroscopic measurements of photolesion formation rates under controlled UV irradiation conditions relevant to the prebiotic environment. In this context, the differential photochemical behavior of thymine and uracil under controlled UV irradiation provides critical insights into the molecular evolution of genetic systems and offers a direct means to determine how a single methyl substitution influences UV response and relative photostability of the two nucleobases (24, 27–34).

It is widely accepted that RNA preceded DNA as the primary carrier of genetic information in the earliest life forms approximately 3.8 to 4.0 billion years ago, a concept known as the “RNA world” hypothesis (35–37). This evolutionary framework suggests that the transition from uracil-containing RNA to thymine-containing DNA occurred to provide early life forms with a more chemically stable molecule for the long-term preservation of genetic information. During this prebiotic era, Earth’s atmosphere lacked a protective ozone layer, allowing intense UV radiation to reach the surface without significant attenuation. Moreover, evidence indicates that the synthesis and early evolution of RNA and its characteristic nucleobase, uracil, occurred predominantly in shallow waters such as ponds and coastal pools, environments directly exposed to solar UV radiation (38–48). Therefore, high-energy UV radiation that effectively damages nucleic acids posed a significant threat to the stability of early genetic polymers. This environmental challenge should have created strong evolutionary pressure favoring molecular structures that could resist photochemical degradation while preserving their essential function as carriers of genetic information. However, this evolutionary transition presents an apparent paradox from a photostability perspective. The eventual replacement of uracil by thymine in DNA appears to have favored a nucleobase that is more susceptible to UV-induced photodamage, as our spectroscopic measurements in this work show that thymine forms these photoproducts more frequently than uracil. In addition, solar UV flux models and theoretical studies of early Earth atmospheric conditions suggest that radiation intensity at the peak wavelength absorbed by thymine was approximately 2.5 times higher than at the absorption maximum of uracil, although the exact flux ratio is model-dependent, and studies of UV transmission in prebiotic shallow waters indicate that nucleobases would have been directly exposed to this differential UVC flux with minimal aqueous attenuation (16, 17, 35, 41, 49, 50). Taken together, these considerations imply that the evolutionary transition from uracil-based RNA to thymine-based DNA may have shifted genetic material into a more intense and potentially more damaging spectral region of solar radiation, thereby raising fundamental questions regarding the selective pressures that governed this molecular substitution. Therefore, understanding and resolving this apparent paradox requires a detailed comparative analysis of the photochemical and photophysical properties of these two nucleobases under controlled UV irradiation conditions.

In the present study, we investigate the photochemical effects of 265 nm UV irradiation on the steady-state absorption, fluorescence, and Raman spectra of thymine and uracil under identical experimental conditions. By comparing their spectral responses and photodamage mechanisms, we aim to provide direct experimental evidence for the differential UV-induced molecular damage of these two nucleobases and determine whether thymine exhibits photochemical properties that could help explain its evolutionary selection in early genetic systems.

Beyond addressing fundamental questions in molecular evolution, the outcomes of this research are expected to provide reference spectroscopic data for future studies on UV-induced nucleobase photodamage and contribute to the development of spectroscopy-based tools for pathogen detection and improved UV disinfection methods for water treatment. Specifically, the comparative spectroscopic characterization of thymine and uracil in both native and photodamaged states provides the molecular-level reference framework necessary for interpreting UV-induced spectral changes in complex biological samples such as whole cells, enabling more accurate and sensitive spectroscopic diagnostics.

## Results

### Absorption Spectra of Thymine and Uracil.

**Fig. 2.**
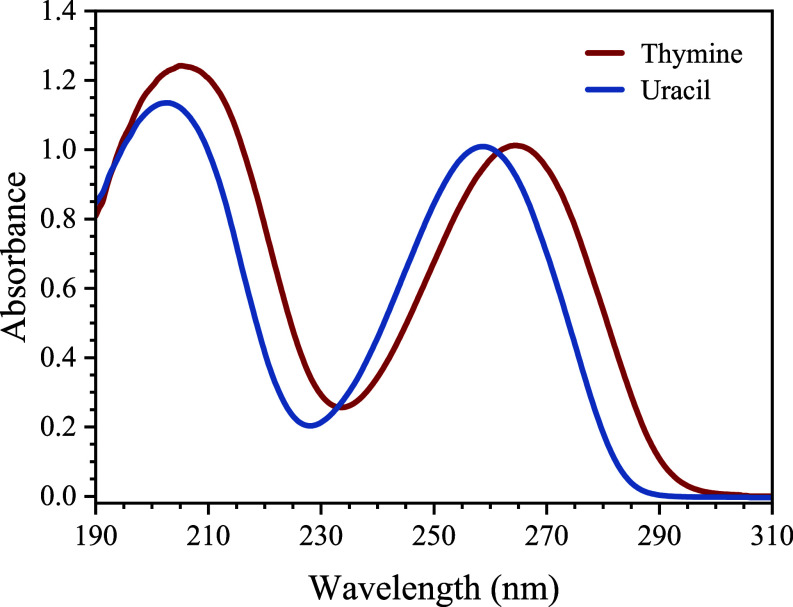
UV absorption spectra of thymine and uracil in aqueous solution (130 µM), showing maxima at 206 nm and 265 nm for thymine and at 203 nm and 259 nm for uracil.

The absorption spectra of thymine and uracil molecules in aqueous solutions are shown in Fig. 2, with thymine exhibiting two absorption band maxima at 206 nm and 265 nm, while uracil exhibits corresponding peaks at 203 nm and 259 nm. The concentrations of thymine and uracil solutions were carefully adjusted to 130 µM so that their corresponding lower-energy absorption maxima at 265 nm and 259 nm reached optical densities of approximately 1, ensuring reliable spectral comparison within the linear detection range and enabling clear visualization of intrinsic spectroscopic differences. The lower-energy absorption peaks near 265 nm for thymine and 259 nm for uracil correspond to π→π* electronic transitions, where an electron is excited from a bonding π orbital (HOMO) to an antibonding π* orbital (LUMO) within the conjugated π system of the pyrimidine ring. This energy is primarily absorbed by the electrons in the C5=C6 double bonds of thymine and uracil, after which the resulting excited molecules predominantly undergo one of three competing pathways: i) internal conversion followed by vibrational relaxation, which dissipates the absorbed energy as heat and returns the excited molecule to its ground state without chemical change; ii) fluorescence emission, wherein the excited molecule releases a photon and returns to the ground state; or iii) photochemical reactions leading to the formation of photoproducts, including CPD and (6-4) photoproducts. The 6 nm redshift observed in thymine’s absorption maximum relative to uracil is attributed to the electron-donating effect of the C5-methyl substituent in thymine, which raises the energy of the π orbitals and consequently reduces the HOMO–LUMO energy gap and shifts the π→π* transition to longer wavelengths. Additionally, the absorption band of thymine near 265 nm has a full width at half maximum (FWHM) of 35.5 nm, approximately 9% broader than the corresponding band of uracil near 259 nm, which has an FWHM of 32.6 nm. The higher-energy maxima near 206 nm (thymine) and 203 nm (uracil) correspond to the second electronic excited state (S_2_). Upon excitation at these higher-energy absorption bands, these molecules can undergo internal conversion, relaxing from the second excited state (S_2_) to the first excited state (S_1_), where most photochemical reactions occur, including the formation of CPDs and (6-4) photoproducts.

### Effect of Concentration on the UV Absorption Spectra of Thymine and Uracil.

**Fig. 3.**
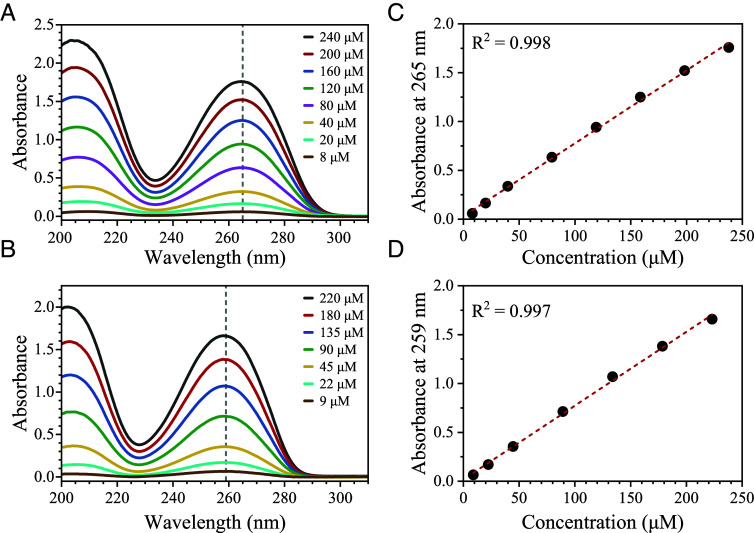
Absorption spectra of thymine (*A*) and uracil (*B*) at varying concentrations, with corresponding Beer–Lambert plots at 265 nm (*C*) and 259 nm (*D*), respectively.

Absorption measurements were conducted to evaluate the spectroscopic response of thymine and uracil to concentration changes. To that effect, Fig. 3 *A* and *B* show the absorption spectra of thymine and uracil in solutions of different concentrations, respectively. At high concentrations, both thymine and uracil exhibit peak flattening in their absorption spectra due to optical density saturation and inner filter effects. For thymine, this effect becomes evident above 240 µM at both the 206 nm and 265 nm bands, while for uracil, similar flattening appears above 220 µM near the 203 nm and 259 nm bands. No spectral shifts were observed with increasing concentration, confirming the absence of π–π stacking interactions or hydrogen-bonded aggregation effects resulting from intermolecular interactions. Conversely, at concentrations below 8 µM, the signal intensity approaches the instrument’s detection limit, resulting in distorted or undetectable spectral features. Despite changes in peak intensity, the FWHM and overall bandwidth of the absorption bands remain effectively constant across the linear concentration range for both nucleobases. Furthermore, Fig. 3 *C* and *D* present the Beer–Lambert plots for thymine and uracil, respectively, showing a clear linear relationship between absorbance at the peak wavelength (265 nm for thymine and 259 nm for uracil) and concentration within the reliable detection range. The molar extinction coefficients were determined to be ~7,700 M^−1^ cm^−1^ for thymine at 265 nm and ~7,850 M^−1^ cm^−1^ for uracil at 259 nm in aqueous solution, with corresponding R^2^ values of 0.998 and 0.997, respectively, indicating strong linearity and confirming compliance with the Beer–Lambert law. These results indicate that the spectroscopic properties of thymine and uracil are governed by intrinsic electronic transitions that are not significantly perturbed by intermolecular interactions at varying concentrations.

### UV-Induced Photodegradation Kinetics of Thymine and Uracil Probed by Absorption Spectroscopy.

**Fig. 4.**
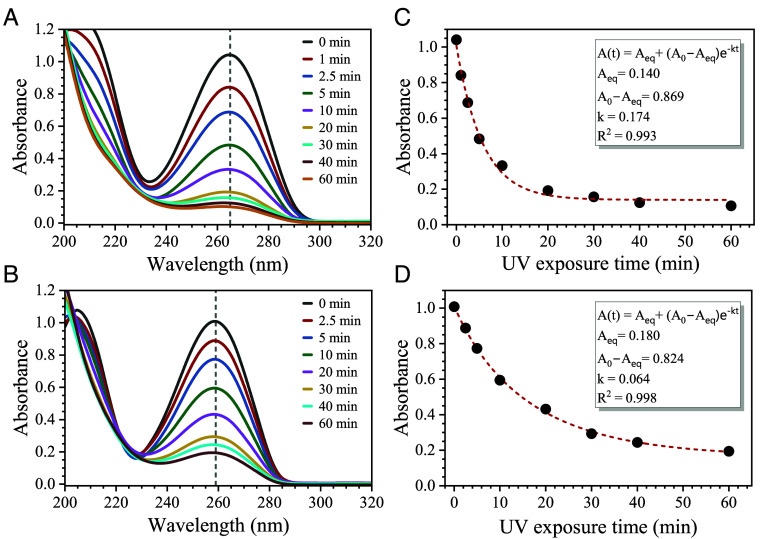
UV-induced photodegradation of thymine (*A*) and uracil (*B*), shown as absorption spectra with increasing exposure time. The corresponding decrease in absorbance as a function of irradiation time is shown at 265 nm for thymine (*C*) and 259 nm for uracil (*D*), fitted using a first-order exponential decay model.

To understand and compare the rates of CPD and (6-4) photoproduct formation in thymine and uracil, aqueous solutions of both pyrimidine bases were subjected to 265 nm UV irradiation (FWHM = 12 nm), exhibiting characteristic photodegradation patterns as illustrated in Fig. 4. For thymine, the absorption peak at 265 nm decreased continuously from an initial optical density (OD) of 1.0 to 0.1 over successive irradiation intervals, after which no further decay was observed (Fig. 4*A*). Similarly, uracil exhibited a reduction in its 259 nm absorption peak from OD = 1.0 to 0.2, reaching a plateau beyond 60 min of UV exposure (Fig. 4*B*). This progressive decay in absorbance is attributed to the photochemical disruption of the C5=C6 double bond in both nucleobases, a key chromophoric site responsible for π→π* transitions in the UV region. Upon irradiation, this bond undergoes photodissociation, initiating the formation of CPDs and (6-4) photoproducts, leading to a loss of the original electronic structure and thus a decline in absorbance at the characteristic wavelengths. The residual absorbance plateau observed for both molecules corresponds to a photostationary equilibrium state, in which the system reaches a dynamic balance between forward photoreactions [CPD and (6-4) photoproduct formation] and reverse photoreactions (dissociation of CPD photoproducts back to monomers), resulting in a small net change in photoproduct concentration despite continued irradiation. This plateau consists of a mixture of unreacted nucleobase molecules that remain photochemically inaccessible due to steric constraints or unfavorable molecular orientations in the frozen state. The lower plateau observed for thymine indicates that a larger fraction of thymine molecules are converted into photoproducts compared to uracil, consistent with the electron-donating effect of the C5-methyl group, which facilitates more efficient molecular packing in the aqueous matrix by providing additional directional intermolecular interactions, specifically CH–π contacts and van der Waals forces that organize neighboring thymine molecules into preferential face-to-face stacking arrangements. This organized assembly positions the reactive C5=C6 double bonds of adjacent thymine molecules at the optimal geometry for photochemical reactions. In contrast, uracil, lacking the methyl substituent, exhibits less directional packing with more heterogeneous molecular orientations. This results in a larger fraction of uracil molecules adopting geometries in which the C5=C6 bonds are either too distant or unfavorably oriented for efficient dimerization. Consequently, even under identical UV irradiation conditions, a greater proportion of uracil molecules remain unreacted and photochemically inaccessible, explaining the higher residual absorbance in the photostationary state.

The decay of the absorption maxima at 265 nm and 259 nm as a function of time is shown in Fig. 4 *C* and *D* for thymine and uracil, respectively. As demonstrated in these figures, nonlinear fitting analysis indicates that the photodegradation of these molecules under UV irradiation follows a first-order exponential decay model of the form:

[1] $$A\left(t\right)=A_{\mathrm{eq}}+\left(A_{0}-A_{\mathrm{eq}}\right)e^{-kt},$$

where *A*(*t*) is the absorbance (optical density) at the respective absorption maximum measured at time *t*, *A*_eq_ is the residual absorbance at the photostationary equilibrium plateau, and *A*_0_ is the initial absorbance before UV exposure, which was approximately 1.0 for both molecules. The effective photodegradation rate constant *k* provides an empirical measure of the net decay rate of the nucleobase absorbance under fixed irradiation conditions, encompassing the combined contributions of the different UV-induced photochemical pathways. The fitted *k* values were determined to be 0.174 min^−1^ and 0.064 min^−1^ for thymine and uracil, respectively. The larger *k* value for thymine indicates a faster degradation process and higher photoreactivity, whereas the smaller *k* value for uracil reflects greater photostability of this nucleobase.

### Fluorescence Emission Properties of Thymine and Uracil.

**Fig. 5.**
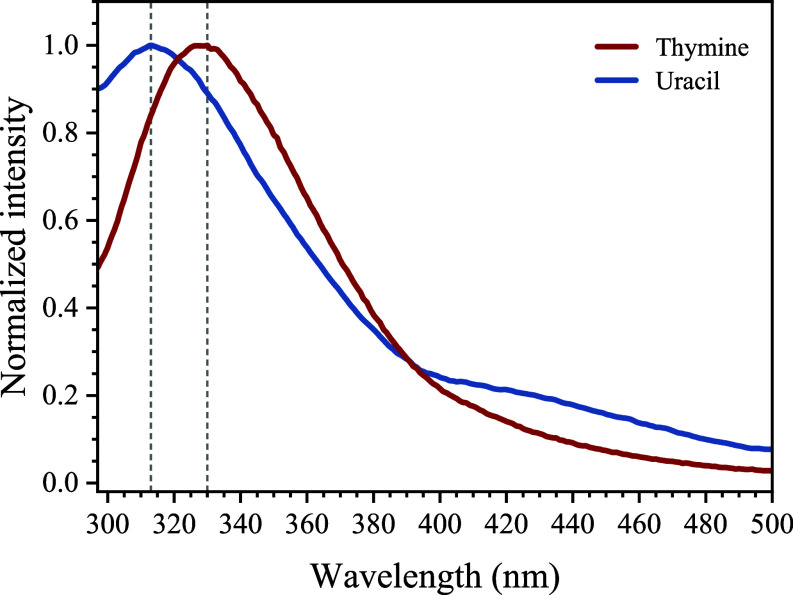
Normalized fluorescence spectra of thymine and uracil excited at their respective absorption maxima (265 nm and 259 nm), showing emission peaks at 330 nm for thymine and 313 nm for uracil.

Upon excitation at their respective absorption maxima (thymine at 265 nm and uracil at 259 nm), both molecules exhibited Stokes-shifted fluorescence emission at 330 nm and 313 nm, respectively, as shown in Fig. 5. Thymine displayed a larger Stokes shift (65 nm vs. 54 nm for uracil) and a narrower intrinsic fluorescence bandwidth (FWHM = 70 nm vs. 100 nm for uracil). The larger Stokes shift of thymine suggests more substantial energy dissipation through vibrational relaxation and solvent reorganization in the excited state prior to emission, while the narrower FWHM suggests emission from a well-defined and more structurally homogeneous excited-state geometry. Together, these spectral features suggest that thymine undergoes a more extensive excited-state reorganization than uracil, meaning that thymine samples a wider range of transient molecular geometries with altered electron density distribution before ultimately relaxing into its well-defined emitting state, potentially creating more opportunities for the molecule to access photochemical reaction pathways leading to an increased probability of CPD and (6-4) photoproduct formation.

This extended relaxation pathway also suggests that thymine may spend more time in the excited state before returning to the ground state. The increased excited-state lifetime enhances the probability of radiative decay, resulting in significantly stronger fluorescence. Consistent with this interpretation, our experimental data show that thymine exhibits a fluorescence intensity approximately 4.5 times greater than that of uracil at their respective emission maxima under identical experimental conditions. Notably, Fig. 5 displays normalized fluorescence spectra to facilitate visual comparison of the spectral profiles and bandwidths; however, the 4.5-fold difference in the measured intensities is not visually apparent due to this normalization.

### Fluorescence Kinetics of UV-Induced Photoproducts in Thymine and Uracil.

**Fig. 6.**
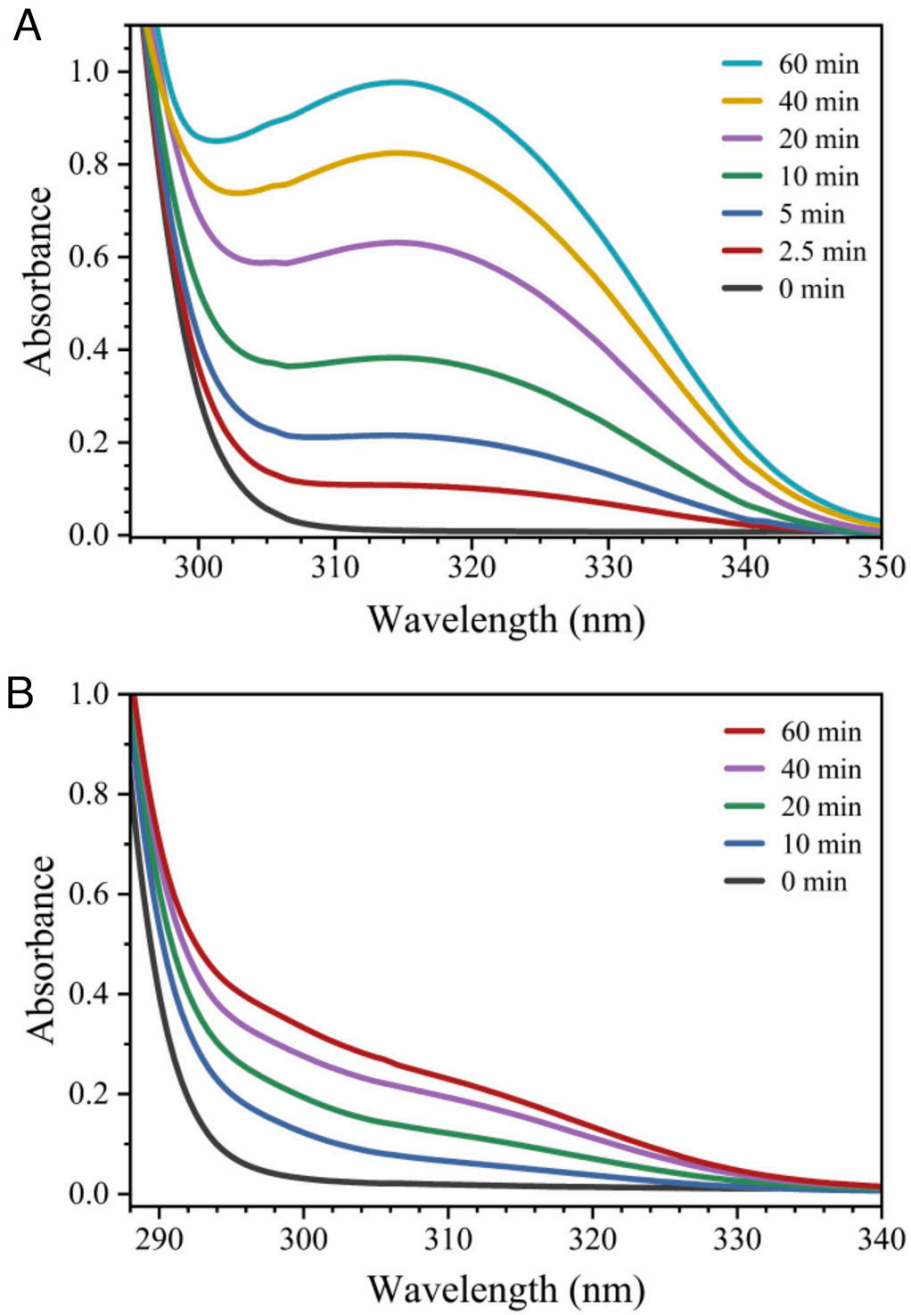
Formation of new photoproduct absorption bands upon UV irradiation of high-concentration aqueous solutions, appearing near 315 nm for thymine (*A*) and 300 nm for uracil (*B*).

**Fig. 7.**
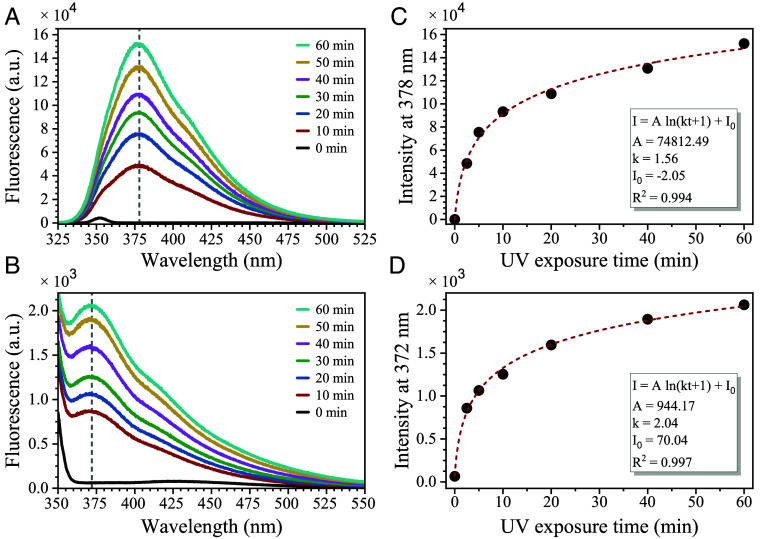
Fluorescence spectra of thymine (*A*) and uracil (*B*) excited at their respective photoproduct absorption band maxima, showing the emergence of new emission peaks at 378 nm and 372 nm, respectively, as a function of UV irradiation time. The corresponding fluorescence peak intensities at 378 nm for thymine (*C*) and 372 nm for uracil (*D*) are plotted versus UV irradiation time and fitted to a logarithmic growth model.

Fig. 6 shows that, upon 265 nm UV irradiation, both thymine and uracil exhibit the emergence of new absorption bands near 315 nm and 300 nm, respectively, which are indicative of the formation of photoproducts. The UV irradiation and absorption measurements were performed at relatively high sample concentrations (8 mM) to achieve sufficient optical density for detecting the emerging photoproduct bands within the spectra. However, because absorption spectroscopy is less sensitive for tracking low-level photoproduct accumulation, the kinetics of band formation were investigated using fluorescence spectroscopy at lower concentrations (130 µM), where the enhanced sensitivity of fluorescence detection enables accurate monitoring of photoproduct evolution. Subsequent excitation of these photoproducts at their corresponding absorption band maxima wavelengths yields distinct fluorescence bands with maxima at 378 nm and 372 nm, as shown in Fig. 7*A* for thymine and Fig. 7*B* for uracil, respectively. Fig. 7 *C* and *D* further show the fluorescence peak intensity of the formed band in thymine and uracil, respectively, as a function of UV exposure time. The results indicate that the band intensity of the photoproducts increases nonlinearly with UV exposure time according to a logarithmic growth model:

[2] $$I\left(t\right)=A\;\ln(kt+1)+I_{0},$$

where A represents the magnitude of the fluorescence intensity change induced by UV irradiation, serving as a scaling factor that defines how strongly the photoproduct fluorescence intensity grows above the baseline with increasing UV exposure time. The parameter k is a rate constant that determines how rapidly photodamage formation proceeds under the applied UV irradiation. The constant $I_{0}$ denotes the initial peak intensity at *t* = 0, prior to UV exposure. Unlike the exponential behavior observed for absorbance decay in Fig. 4, which reflects the photoreversible nature of CPD formation and dissociation, this logarithmic fluorescence growth captures the accumulation of largely nonreversible photoproducts, such as pyrimidine (6-4) pyrimidone lesions. These nonreversible lesions continue to form without undergoing significant photoreversal, leading to a monotonic increase in fluorescence with a progressively decreasing growth rate due to the depletion of unreacted nucleobases and geometric constraints that limit further dimerization within the irradiated sample. This interpretation, inferred from the kinetic behavior of the formed photoproducts, is in good agreement with previous studies that directly characterized (6-4) photoproducts as largely nonreversible lesions and established their absorption bands near 300 to 320 nm as well as distinct fluorescence emission near 380 nm, consistent with the bands observed here for thymine and uracil (6-4) photoproducts (51–55).

In thymine, the fluorescence intensity of the emerging photoproduct band increases with irradiation time according to the logarithmic growth model (Eq. **2**), with fitting parameters A= 74,812, $k=\;1.56\;\min^{-1}$, $I_{0}=-2.05$, and a strong correlation (R^2^ = 0.994). Under identical experimental conditions, the fluorescence growth kinetics of uracil also follow the logarithmic model with A= 944, $k=\;2.04\;{\text{min}}^{-1}$, $I_{0}=70.04$, and R^2^ = 0.997. The lower photoproduct formation rate constant (k) observed for thymine, combined with its larger amplitude parameter (A= 74,812), indicates that thymine accumulates photoproducts more gradually and reaches a significantly higher fluorescence intensity at longer irradiation times compared to uracil. The larger fluorescence amplitude observed for thymine photoproducts is consistent with their approximately threefold greater absorbance at 315 nm compared to uracil photoproducts at 300 nm, as shown in Fig. 6. This enhanced fluorescence of thymine photoproducts is further supported by the work of Blais et al., which demonstrated that the presence of a C5-methyl group on the pyrimidone ring significantly increases the fluorescence quantum yield of (6-4) photoproducts (52).

### Raman Spectroscopic Signatures and Kinetics of UV-Induced Photodamage in Thymine and Uracil.

**Fig. 8.**
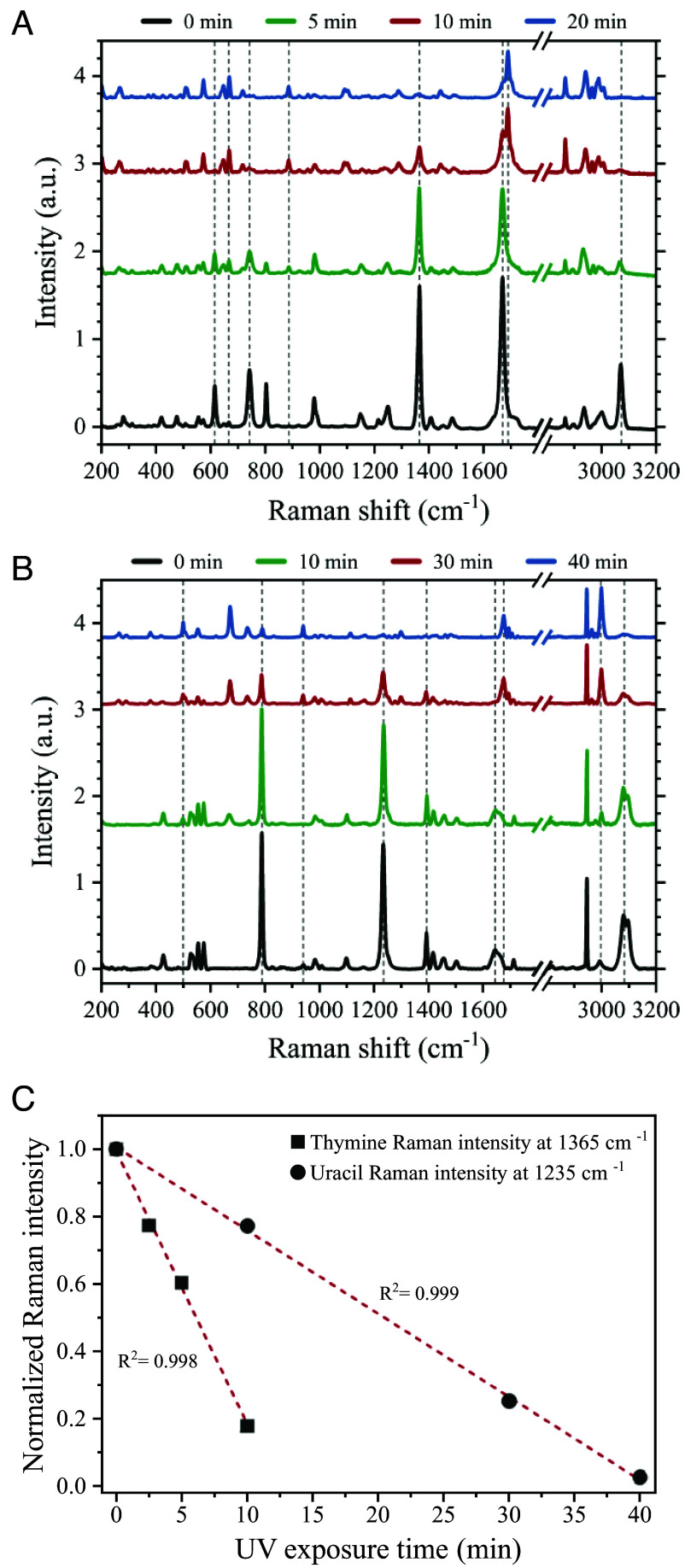
Raman spectra of thymine (*A*) and uracil (*B*) recorded before and after exposure to UV irradiation. Linear decay of the Raman peak intensity at 1,365 cm^−1^ for thymine and 1,235 cm^−1^ for uracil as a function of UV exposure time (*C*), reflecting the disruption of the pyrimidine ring and photoproduct formation.

The steady-state Raman spectra of thymine and uracil molecules are shown in Fig. 8 *A* and *B*, respectively, and were acquired before and after various durations of 265 nm UV irradiation in the frozen state. The C5=C6 double bond stretching vibrations, coupled to the adjacent carbonyl group, appear at 1,670 cm^−1^ for thymine and 1,644 cm^−1^ for uracil. These bands exhibit notable sensitivity to UV irradiation, with progressive reduction in peak intensity and frequency shifts indicating the dissociation of the π-bond system and the subsequent formation of covalent σ-bonds within CPD and (6-4) photoproducts. Similarly, the prominent ring-breathing modes centered at 742 cm^−1^ for thymine and 789 cm^−1^ for uracil show a continuous intensity decay as a function of UV irradiation time, indicating disruption of π-electron conjugation as the planar ring system becomes distorted upon cyclobutane and oxetane ring formation. Furthermore, the band observed near 1,365 cm^−1^ for thymine, corresponding to the CH_3_ umbrella bending mode, and the distinct uracil band near 1,235 cm^−1^, arising from an in-plane stretching mode coupled to N–H and C–H bending, both exhibit a progressive intensity decay upon UV irradiation, reflecting the loss of the monomeric ring vibrational character as a result of C5=C6 π-bond disruption and the formation of photoproducts. To that effect, Fig. 8*C* shows the Raman peak intensities of these characteristic bands versus UV irradiation time, revealing an approximately linear decay relationship that quantitatively measures the photoreaction kinetics of these modes under the employed experimental conditions. The slope of this linear decay is greater for thymine than for uracil, indicating faster disruption of the C5=C6 double bond in thymine and, consequently, a higher rate of photoproduct formation under identical irradiation conditions.

Differentiation between the two photodamage pathways was achieved by monitoring pathway-specific spectral features emerging upon UV exposure. CPD formation was identified by the appearance of spectral bands characteristic of the newly formed cyclobutane ring. In thymine, a new band formed at 887 cm^−1^ assigned to the cyclobutane ring breathing mode, accompanied by intensification of the bands at 649 and 666 cm^−1^ attributed to cyclobutane ring deformation vibrations. Correspondingly, uracil exhibited analogous CPD markers including a new band at 940 cm^−1^ assigned to cyclobutane ring breathing and intensification at 480 cm^−1^ arising from cyclobutane ring deformation. Signatures of (6-4) photoproduct formation were also observed in both molecules, with thymine displaying a new band at 1,690 cm^−1^ and uracil at 1,676 cm^−1^, both assigned to pyrimidone carbonyl stretching in the (6-4) photoproduct. These pathway-specific spectral signatures, together with the diminishing C5=C6 and ring-breathing modes, serve as reliable diagnostic markers for quantitative assessment and mechanistic differentiation of UV-induced photodamage in pyrimidine nucleobases.

To assess the potential relaxation pathways available to each nucleobase following UV photoexcitation, we compared the total count of Raman-active vibrational modes between uracil and thymine. According to vibrational theory, nonlinear molecules possess 3*N*−6 normal modes, where *N* represents the number of atoms. Based on their molecular formulae, thymine (C_5_H_6_N_2_O_2_, 15 atoms) and uracil (C_4_H_4_N_2_O_2_, 12 atoms) are predicted to exhibit 39 and 30 fundamental vibrational modes, respectively. In our experimental spectra acquired prior to UV irradiation, thymine exhibited 35 distinct Raman peaks, while uracil displayed 32 peaks across the measured spectral range, as shown in Fig. 8 *A* and *B*, respectively. The observation of more peaks than theoretically predicted for uracil suggests contributions from overtone and combination bands, whereas thymine’s lower observed count indicates that some fundamental modes are either too weak to detect, spectrally overlapped, or fall outside the measured frequency range. Notably, this difference in vibrational mode distribution becomes more pronounced in the low-frequency region (<1,000 cm^−1^), where thymine exhibits 19 peaks compared to 12 in uracil. Low-frequency modes promote efficient thermal relaxation because their smaller energy quanta facilitate mode-to-mode coupling, and their higher density of states increases vibrational transition probability. Low-frequency molecular vibrations also couple more effectively with lattice phonons and solvent modes, enabling efficient energy dissipation to the environment. These characteristics of thymine provide additional pathways for dissipating UV-deposited energy into vibrational motion rather than photochemical bond rearrangement.

## Discussion

### Evolutionary Implications of the Differential UV Responses of Thymine and Uracil.

In this work, the photophysical and photochemical responses of thymine and uracil to UV irradiation were examined under conditions designed to mimic the primordial solar UVC spectrum, using an LED whose emission is centered at 265.3 nm, delivering a photon flux approximately 2.5 times greater at the absorption maximum of thymine (265 nm) than at that of uracil (259 nm). Thymine and uracil exhibit comparable molar extinction coefficients at their respective band maxima, but thymine’s absorption band is ~9% broader, leading to enhanced spectral overlap with the incident radiation.

Steady-state fluorescence measurements reveal that thymine exhibits a larger Stokes shift and a narrower emission bandwidth, suggesting that thymine undergoes more extensive excited-state reorganization and samples a wider range of transient molecular geometries before relaxing into a well-defined emitting state. This extended excited-state reorganization potentially increases opportunities for accessing photochemical reaction pathways that lead to the formation of photoproducts. These results are in good agreement with previous computational studies proposing the existence of a potential energy barrier on the first excited-state energy surface of the thymine molecule (56–61). This barrier hinders the excited molecule from efficiently reaching the S_1_/S_0_ conical intersection that mediates internal conversion to the ground state, thereby prolonging its residence time in the excited state and increasing the probability of both competing photochemical reactions and radiative decay via fluorescence before thermal relaxation can occur. Notably, although thymine possesses a greater density of low-frequency vibrational modes that would otherwise facilitate efficient thermal energy dissipation, the presence of this excited-state barrier effectively negates this intrinsic advantage by preventing the excited molecule from accessing the conical intersection where vibrational cooling to the ground state would occur. In alignment with this interpretation, our experimental results demonstrate that thymine displays a fluorescence intensity roughly 4.5 times greater than that of uracil at their respective emission maxima under identical experimental conditions. Furthermore, our data show that thymine forms photoproducts more readily under the same UV irradiation conditions, as evidenced by the faster loss of UV absorption intensity and the more rapid attenuation of key Raman bands.

Therefore, these results present a compelling paradox: Thymine, the nucleobase that ultimately prevailed in DNA over its simpler analog uracil, appears to have evolved not only to form photodamage more readily than uracil but also to have its absorption band both shifted and broadened into a spectral region where the primordial solar UVC flux was substantially higher.

Resolving this paradox therefore requires considerations that extend beyond the intrinsic photostability of the isolated nucleobases themselves, encompassing higher-order structural, environmental, and evolutionary factors that modulate damage formation and self-repair. In this context, our data support the “molecular sunscreen” hypothesis, which proposes that nucleobases were evolutionarily tuned to absorb within the more intense spectral windows of the ozone-free primordial UV environment, functioning as sacrificial UV absorbers by capturing UV radiation and localizing photodamage within the bases to protect more vulnerable components of nucleic acids, such as the sugar-phosphate backbone that is approximately two orders of magnitude more susceptible to photodamage and whose direct excitation would more readily lead to strand breaks (57, 62).

In addition, nucleobases appear to have been evolutionarily tuned to ensure that the photodamage they inevitably accumulate is constrained to a narrow set of photolesions that are intrinsically more amenable to self-repair through nonenzymatic mechanisms that would have been operative long before the emergence of sophisticated repair enzymes (63–65). This self-repair mechanism operates under UVC irradiation, implying that primordial nucleic acids could exploit the same intense solar flux responsible for their damage to drive lesion reversal. To that effect, our kinetic data provide direct experimental support for this evolutionary interpretation. Although thymine undergoes more rapid overall photodamage formation than uracil, as evidenced by its faster UV absorbance decay and more rapid attenuation of Raman signatures, it exhibits a notably lower rate constant for irreversible (6-4) photoproduct accumulation ($k=\;1.56\;\text{vs}.\;2.04\;{\text{min}}^{-1}$ for uracil), suggesting that thymine preferentially funnels its photodamage into the reversible CPD pathway rather than the irreversible (6-4) pathway.

These observations suggest that the canonical bases were not optimized to minimize photodamage in an absolute sense, but rather to balance inevitable damage formation with its safe localization and efficient reversibility in a way that maximized the long-term viability of nucleic-acid-based genetic systems in a high-UVC environment.

Beyond the photochemical perspective, a complementary biochemical rationale based on replication fidelity also supports thymine’s selection over uracil in DNA. Spontaneous hydrolytic deamination of cytosine residues within DNA converts them to uracil, and if uracil were itself a canonical base of DNA, such mutagenic lesions would be indistinguishable from native bases and could not be selectively repaired. The use of thymine as the canonical base therefore allows repair enzymes such as uracil-DNA glycosylase (UDG) to recognize and excise any uracil appearing in DNA as a mutagenic lesion rather than a normal constituent (43, 66, 67). However, this mechanism relies on sophisticated repair enzymes that would not have been available in the prebiotic era (68). Therefore, the distinct photochemical response of thymine compared to uracil documented in the present study, which operates through nonenzymatic self-repair mechanisms, likely represents an earlier and more primitive selective pressure, with deamination-based replication fidelity emerging as a complementary and reinforcing factor at a later stage of molecular evolution.

### Applications in Pathogen Detection and UV Disinfection Monitoring.

Recently, spectroscopic techniques have emerged as an alternative to traditional pathogen detection methods owing to the fact that they are significantly faster, highly sensitive at low concentrations, noninvasive, and relatively low-cost (69–73). In our earlier studies (74, 75), we have demonstrated the development of handheld spectroscopic devices capable of recording absorption, emission, and Raman spectra of bacterial samples in situ within minutes. However, interpreting such multicomponent spectra of the whole cell and quantifying UV-induced damage both require fundamental reference data from the underlying molecular components and their photoproducts. Hence, establishing detailed spectroscopic properties of pyrimidine nucleobases in both native and photodamaged states provides an essential foundation for pathogen detection and UV disinfection monitoring.

The comparative spectroscopic characterizations of thymine and uracil presented in this work provide a molecular-level framework for interpreting UV-induced spectral fingerprints in complex biological samples and for the optimization of UV-based diagnostic and disinfection systems. By isolating the individual pyrimidine bases and quantifying their absorption, fluorescence, Raman, and photodegradation behaviors using a 265 nm LED, we establish how subtle differences in nucleobase structure translate into measurable spectroscopic signatures and distinct UV damage kinetics. These nucleobase-resolved properties facilitate the deconvolution of multicomponent spectra obtained from whole cells and allow for the conversion of spectral changes into quantitative metrics of UV dose and photodamage accumulation. Furthermore, the distinct optical fingerprints of thymine and uracil enable spectroscopic differentiation between DNA-dominated and RNA-dominated systems, a key requirement for UV-based diagnostic and disinfection technologies.

Raman spectroscopy adds a critical advantage for in situ pathogen detection and UV disinfection monitoring because it provides chemically specific markers that are less ambiguous than changes in the broadband absorption and fluorescence spectra. In particular, the thymine-specific CH_3_ fingerprint band near 1,365 cm^−1^ in Fig. 8*A*, assigned to the CH_3_ umbrella bending mode, serves as a unique marker for thymine that is absent in uracil and can therefore distinguish DNA from RNA in spectroscopic analysis. The intensity of this peak decreases linearly upon UV irradiation, as the disruption of the C5=C6 π-bond changes the C5 carbon hybridization from sp^2^ to sp^3^, thereby significantly reducing the Raman polarizability of the methyl group, even though the methyl group itself remains structurally intact. Our experimental results also establish a spectroscopic basis to differentiate between CPD and (6-4) product formation upon UV irradiation, based on their characteristic Raman spectral bands. The emergence of cyclobutane ring breathing modes (887 cm^−1^ for thymine, 940 cm^−1^ for uracil) provides direct spectroscopic evidence for CPD formation, while the appearance of pyrimidone C=O stretching bands (1,690 cm^−1^ for thymine, 1,676 cm^−1^ for uracil) specifically indicates (6-4) photoproduct accumulation. This pathway-specific discrimination is valuable for disinfection monitoring because, in many pathogens, CPDs are repaired more efficiently through photoreactivation than (6-4) photoproducts when cells are subsequently exposed to visible or UVA light, potentially enabling pathogen recovery. Consequently, the CPD-to-(6-4) ratio becomes a key determinant of whether UV-treated pathogens will remain inactivated when posttreatment light exposure occurs.

By means of absorption spectroscopy, we have characterized the parent absorption band maxima (265 nm for thymine, 259 nm for uracil) and established a first-order exponential kinetic model (Eq. **1**) that quantitatively describes the decay of these bands under UV irradiation. Concurrently, weak absorption bands emerge at ~315 nm and ~300 nm for thymine and uracil, respectively, indicating photoproduct accumulation. While the photoproduct absorption bands are weak, fluorescence spectroscopy provides a far more sensitive diagnostic signal at longer wavelengths, enabling quantification of photoproduct concentrations too low to be detected by absorption spectroscopy. Upon UV irradiation, excitation of the newly formed long-wavelength photoproduct absorbers (~315 nm in thymine and ~300 nm in uracil) yields distinct photoproduct fluorescence bands that emerge at 378 nm for thymine and 372 nm for uracil, with growth kinetics following a logarithmic growth model (Eq. **2**) that enables a quantitative correlation between fluorescence intensity and UV dose. Furthermore, the substantially higher fluorescence intensity of thymine photoproducts (approximately 79 times more intense than that of uracil) implies that fluorescence-based damage detection may be inherently more sensitive for DNA-containing samples than for RNA-containing samples, a consideration relevant to assay design in pathogen monitoring applications.

## Experimental

### Sample Preparation.

The thymine used in this study was purchased from Tokyo Chemical Industry Co., Ltd. (TCI; catalog no. T0234), with a stated purity exceeding 98%, and was used as received. A concentrated stock solution was prepared by adding 50 mg of thymine to 50 mL of distilled water to achieve a concentration of 1 mg/mL (8 mM). Upon mixing, some colloidal aggregates were formed due to hydrophobic stacking interactions between nucleobase molecules and the aqueous environment, indicating incomplete dissolution of the nucleobases. To ensure homogeneous solutions without colloidal artifacts, the samples were subjected to shaking at 1,000 rpm for 3 h. The samples were then visually inspected for clarity and further verified spectrophotometrically at 600 nm with absorbance values below 0.005 to confirm the absence of colloidal aggregates. The second compound, uracil, was procured from Sigma-Aldrich (catalog no. U0750) with purity exceeding 99% and was prepared following the same protocol as thymine to ensure consistency. Water was used as the solvent throughout the experiments to mimic the biological environment of nucleic acids while remaining optically transparent in the UV region of interest above 200 nm, making it an optimal medium for spectroscopic measurements without solvent interference. All solutions were prepared in a dark environment under dim red light at room temperature (23 ± 1 °C) to prevent photodegradation. To perform UV irradiation experiments, samples were prepared at a final concentration of 130 µM for both thymine and uracil, corresponding to an optical density of 1 at 265 nm and 259 nm, respectively, unless otherwise stated. Solutions were maintained in sealed airtight containers at ~4 °C for a maximum of 48 h before exposure to UV radiation. To establish a linear calibration curve and ensure accurate molar absorptivity determination, standard solutions were serially diluted across a seven-point concentration range from 240 to 8 µM, while adhering to the Beer–Lambert law.

### UV Irradiation.

After preparation, 0.7 mL aliquots of the nucleobase samples were transferred into a 2 × 2 × 2 cm quartz cuvette and subsequently frozen at −15 °C using a temperature-controlled laboratory freezer to ensure solid-state conditions necessary for the time-dependent irradiation processes. The irradiation of pyrimidine nucleobases was performed in the frozen state because when aqueous solutions of nucleobases are cooled below freezing, the growing hexagonal ice crystals expel pyrimidine molecules from the solvent, creating highly concentrated regions where intermolecular distances between solute molecules are significantly reduced. Furthermore, the rigid environment of the frozen matrix further constrains vibrational and rotational motion of solute molecules, which leads to minimizing the competing thermal relaxation pathways, effectively increasing the quantum yield of photoproducts. This arrangement closely mimics the geometric proximity and stacking of nucleic bases in nucleic acid strands, where photoreactions such as dimer formation can occur between adjacent pyrimidine bases upon UV irradiation, although the frozen, close-packed environment is a controlled model system that does not capture the full structural and chemical complexity of nucleic acids under physiological or prebiotic aqueous conditions (76–82).

**Fig. 9.**
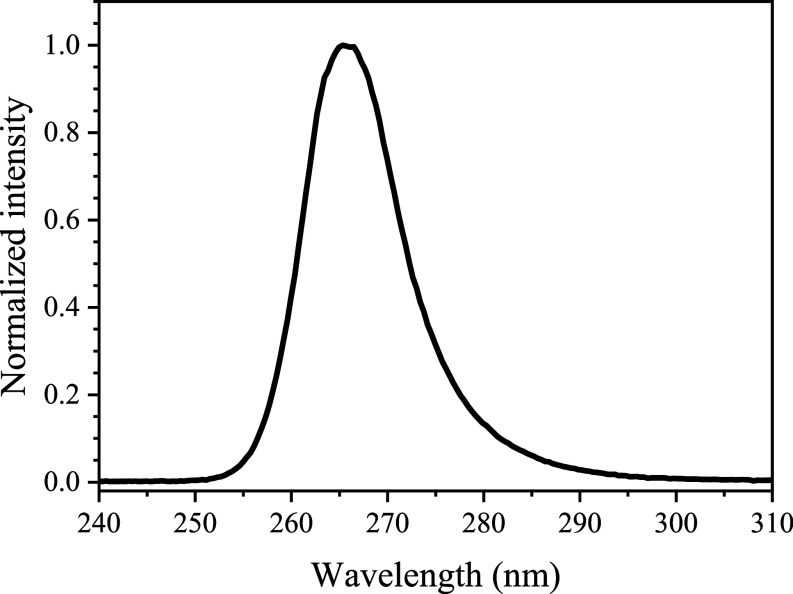
Emission spectrum of the 265 nm LED used for sample irradiation.

Frozen nucleobase samples were then irradiated using a UVC LED (Stanley-ZEUBE265) with peak emission at 265.3 nm and an FWHM of 12 nm, as shown in the emission spectrum in Fig. 9. A narrowband UVC LED was used instead of a broad-spectrum UV lamp in order to isolate the effect of direct photodamage while minimizing other effects, such as indirect oxidation. The wavelength of 265 nm was chosen because it lies near the absorption maxima of the nucleobases and represents a biologically and technologically relevant UVC wavelength. Notably, the relative emission intensity of this LED at 265 nm compared to 259 nm is consistent with the ratio estimated from solar UV flux models of the prebiotic era, in which the radiation intensity at thymine’s absorption peak (~265 nm) was approximately 2.5 times greater than at uracil’s absorption peak (~259 nm). This correspondence makes the chosen wavelength particularly relevant for investigating the differential photochemical responses of these two nucleobases and the role of UV irradiation in the evolutionary transition from uracil-based RNA to thymine-based DNA systems (49, 50). To ensure uniform irradiation, the LED was positioned vertically at a fixed distance of 10 cm above the quartz cell, with a beam angle of 120°. During the entire irradiation procedure, the power density of the LED was maintained constant at 0.6 mW/cm^2^, as measured directly at the sample plane using a DET10A2 silicon-based optical power meter. A thermometer placed adjacent to the cuvette confirmed that the temperature at the sample location remained at −15 °C with a maximum deviation of ±0.7 °C throughout each irradiation period and during storage of the control samples, indicating negligible heating from the UV source and consistent thermal conditions across all experiments.

### Spectroscopic Measurements.

The absorption, fluorescence, and Raman spectra were measured in a dark environment under dim red light at room temperature, before and after each UV irradiation period. This precaution was taken to prevent ambient light from inducing unintended photochemical reactions, such as the photodissociation of accumulated photoproducts. For each spectral measurement, at least three independent spectral acquisitions were performed and averaged to improve the signal-to-noise ratio unless otherwise stated. For irradiated samples, measurements were performed immediately after thawing to room temperature (23 ± 1 °C). For all spectroscopic techniques, nonirradiated control samples were prepared and measured in parallel with irradiated samples under identical conditions at each time point. These control samples exhibited less than 5% variation in spectral intensity across all measurements, confirming that the observed spectral changes in irradiated samples were caused solely by UV irradiation rather than spontaneous degradation or instrumental drift.

UV absorption spectra were recorded using a Shimadzu UV-1601 spectrophotometer with a double-beam optical configuration and silicon photodiode detectors, covering a spectral range of 190 to 1,100 nm, although our studies focused primarily on the far UV region (190 to 320 nm) where nucleobase absorption is most prominent. All absorption spectra were recorded at a scan speed of 370 nm/min, and data sampling was performed every 0.2 nm. A pair of quartz cuvettes with a 10 mm pathlength and 0.7 mL volume, each having two polished optical windows and a high UV transmission of more than 85% at 200 nm, were utilized for both sample and reference cells. Prior to each measurement session, the spectrophotometer was allowed to warm up for 30 min to ensure lamp stability before calibration and then the baseline was corrected using identical cuvettes filled with distilled water to account for solvent effects and instrumental fluctuations.

Fluorescence spectra were recorded using a Shimadzu RF-6000 fluorescence spectrophotometer and a reduced-volume quartz fluorescence cuvette having external dimensions of 10 mm × 10 mm × 40 mm, a 2 mm internal chamber width, 0.7 mL volume, and four transparent optical windows. All emission spectra were recorded over the range 320 to 600 nm, using excitation wavelengths of 315 nm for thymine and 300 nm for uracil, unless otherwise stated, with a scan speed of 600 nm/min and a data interval of 0.1 nm. Excitation and emission slit widths were both set to 10 nm for thymine and uracil, with neutral-density (ND) optical filters inserted in the emission beam path for thymine measurements to compensate for its stronger fluorescence signal and prevent detector saturation. The attenuation introduced by these filters was explicitly included in the data processing, so the reported thymine fluorescence intensities reflect the true signal levels.

Furthermore, a Horiba XploRa™ PLUS Raman spectrometer coupled to a microscope and a 532 nm laser with a nominal output power of 25 mW was utilized to obtain the Raman spectra of thymine and uracil before and after each irradiation dose. The laser power delivered at the sample was measured to be 6.6 mW using a calibrated power meter. For each Raman measurement, a 10 μL aliquot of the aqueous nucleobase solution (both nonirradiated controls and irradiated samples at each dose) was placed on an aluminum mirror and dried prior to spectral acquisition. To enhance spectral intensity and improve the signal-to-noise ratio, a 10× microscope objective and 2,400 g/mm grating were used. The wavenumber axis was calibrated using the fundamental Raman band of a silicon wafer at 520.7 cm^−1^. All spectra were acquired with an integration time of 15 s. For each irradiation dose, 10 individual spectra were collected from each of five independently prepared dried films, and the resulting 50 spectra were averaged to produce the final spectrum, which was subsequently baseline-corrected prior to analysis. This approach normalizes Raman intensities through fixed analyte mass and constant acquisition parameters, while multispot, multifilm averaging minimizes the influence of film inhomogeneity on the reported band intensities. No systematic changes in band positions, relative intensities, or the appearance of new peaks were observed within the 10 accumulated spectra or across the five samples, indicating that laser-induced heating and photochemical alteration during Raman measurements were negligible under our experimental conditions.

## Data Availability

All study data are included in the main text.
